# Oxidative Decomposition of Poly(phenylene sulfide) Composites Under Fast Elevation of Temperature

**DOI:** 10.3390/polym17111560

**Published:** 2025-06-03

**Authors:** Aurélie Bourdet, Yann Carpier, Eric Dargent, Benoit Vieille, Nicolas Delpouve

**Affiliations:** INSA Rouen, UNIROUEN, Normandie Univ, CNRS, GPM UMR6634, 76000 Rouen, France; aurelie.bourdet@ensam.eu (A.B.); yann.carpier@latecoere.aero (Y.C.); eric.dargent@univ-rouen.fr (E.D.); benoit.vieille@insa-rouen.fr (B.V.)

**Keywords:** thermal degradation, oxygen, thermoplastic composites, high-rate TGA, TGA–FTIR

## Abstract

The thermal resistance of carbon fiber–reinforced poly(phenylene sulfide) to harsh oxidative conditions is investigated through thermogravimetric experiments performed in an oxygen atmosphere. While these materials usually show great resistance against thermal decomposition in a nitrogen atmosphere, the experiments in oxygen reveal the total decomposition of both the matrix and the carbon fibers. The Gram–Schmidt signal, obtained by coupling thermogravimetric analysis in standard conditions with Fourier-transform infrared spectroscopy, exhibits multiple events, evidencing that the decomposition proceeds through distinct stages. The first step characterizes the char formation, while the second relates to its oxidative decomposition. A third step, only observed for composites, is interpreted as the signature of the oxidative decomposition of carbon fibers. To mimic the sudden elevation of temperature encountered during a fire, the analyses are performed at rates of up to 500 K min^−1^. These specific experimental conditions reveal a complex dependence of the thermogravimetric signature on the heating rate. Independent of the atmosphere, nitrogen or oxygen, the characteristic temperature of decomposition follows a bell-shape trend, resulting from the combination of lag effects and thermal-conductivity limitations. Additionally, the increase of the heating rate causes the Gram–Schmidt signal to evolve toward a broad peak with indistinct events. To investigate whether these changes affect the decomposition products, the infrared spectra, continuously recorded to probe the whole decomposition, are compared with those from the database. The char formation is characterized by the production of sulfur dioxide, while carbon dioxide is the main product emitted during both char and fiber oxidative decomposition. Owing to the merging of the decomposition stages, sulfur-dioxide detection is partly supplanted by that of carbon dioxide under fast elevations of temperature.

## 1. Introduction

Owing to their recyclability, low water uptake, and excellent impact toughness, thermoplastic matrices have recently raised interest for replacing thermosets polymers in high-performance composites for aeronautic applications [1,2]. Numerous studies have been dedicated to characterizing the properties of poly(phenylene sulfide) (PPS) [3,4,5,6,7,8,9], poly(ether-ether-ketone) (PEEK) [10,11,12,13,14,15], poly(ether-ketone-ketone) (PEKK) [16,17,18,19], and polyetherimide (PEI) [20,21], among others. The aeronautic field constitutes an attractive market for these materials since the use of composites allows the reduction of the weight of the aircraft while maintaining its structural integrity [22]. Fire represents a major risk in the aeronautic industry; the resistance of these materials against thermal decomposition is regularly tested, thanks to thermogravimetric analysis (TGA) investigations. This technique generates information about the temperature domains within which the decomposition steps occur, and helps to gain knowledge regarding the decomposition mechanisms, which allows experimenters to anticipate the degassing of volatile sub-products that may be harmful or feed the fire [23].

High-performance thermoplastic composites are generally characterized by their strong thermal resistance. Regarding the reinforcement, carbon fibers exhibit a sublimation temperature above 3000 °C in an inert atmosphere [24]. Regarding the matrix, its decomposition leads to the formation of a porous carbon mass residue named «char» [25], which can constitute more than 50% of the initial mass. The char protects the material, by favoring flame retardation [26,27]. Moreover, its formation consumes energy and its decomposition is slow [28]. For instance, the anisothermal degradation of PPS, analyzed by TGA in a neutral atmosphere [29], reveals that the char only loses 11% of its mass between 600 and 900 °C. The matrices of PPS, PEEK, PEKK, and PEI all exhibit in their chemical structure some aromatic rings that constitute the molecular bricks from which the char is built, which contributes to their better thermal stability in comparison with other polymers.

To identify the volatile products released during the thermal decomposition, thermal analyses investigations are frequently coupled with other analytical techniques [30,31,32]. As examples, the couplings of TGA with mass spectroscopy (TGA–MS) [33], and Fourier-transform infrared spectroscopy (TGA–FTIR) [34], allows the investigation of the mechanisms lying behind the thermal decomposition of PPS. From the identification of thiophenol among the emitted products, it was deduced that depolymerization is the main mechanism of PPS decomposition, when it occurs in an inert atmosphere.

However, there is a notable difference regarding the elevation of temperature within the material that results from fire on the one hand, or from standard TGA experimental conditions on the other. As shown by Carpier et al. [29], when carbon fiber-reinforced PPS (CF/PPS) faces a fire of medium intensity, its temperature rises at least ten times faster than the conventional rates applied during TGA investigations. By bridging this gap, it should be possible to uncover new information benefiting the fields for which fire safety is a major concern.

In a previous work [35], TGA–FTIR has been performed at rates ranging from 5 to 500 K min^−1^, to study the thermal decomposition of PPS and CF/PPS in a nitrogen atmosphere. At conventional rates, the formation of thiophenol has been recorded, confirming the PPS matrix decomposition by a depolymerization mechanism. In contrast, at rates higher than 100 K min^−1^, it has been observed that the formation of benzene becomes predominant, suggesting that the decomposition occurs by a random chain scission. Thus, two competing mechanisms have been identified, whose relative importance seemingly depends on the heating rate.

The case under scrutiny involves thermal decomposition in oxygen. Indeed, despite their intrinsic thermal resistance, both thermostable matrices and carbon fibers are sensitive to oxidation. Previous TGA studies performed on CF/PPS suggest its total decomposition at 950 °C under air [36], and at 750 °C under oxygen [29], for conventional heating rates (20 and 10 K min^−1^ respectively). Obviously, the increase of the oxygen concentration reduces the thermal resistance of the matrix and fibers. Therefore, the analyses performed in an oxygen atmosphere would help predict the most critical scenario, by delimiting the lower limit for the composite thermal properties.

Among the thermostable polymers and composites that were subjected to high–rate TGA under nitrogen, PPS and CF/PPS stand out, being the only materials to yield a higher amount of char with the increase of the heating rate [35]. Minimizing mass loss is generally requested to maintain the structural stability of a component for a longer time, which could favor PPS and CF/PPS for specific applications. Their decomposition at high rates should now be tested in reactive conditions. Thus, the goal of this study is to gain additional information regarding the thermal properties of PPS and CF/PPS through analyses performed in an oxygen atmosphere. In the first part, general features regarding the decomposition under oxygen were acquired from conventional dynamic and isothermal TGA investigations. Then, the influence of the heating rate on the TGA signature was investigated. Eventually, the decomposition mechanisms were investigated at conventional and fast heating rates, by means of TGA–FTIR experiments.

## 2. Experimental

### 2.1. Materials

Poly(phenylene sulfide) (PPS) Fortron 0214 Celanese^®^ grade was purchased by Ticona^®^, Puteaux, France). The carbon reinforcement is a balanced fabric with a satin pattern of 5 (T300 3K 5HS, Toray, Paris, France) [29]. The volume fraction of fibers is 50%, which corresponds to a matrix mass content of 42%. The composites design consists of 7–ply laminates with an angle ply {±45}7 stacking sequence. For comparison purposes, several properties of PEEK composites reinforced with a continuous carbon fabric (Tenax^®^-E HTA40 3 K), structured in a 5-harness satin weave, were also investigated.

### 2.2. Thermogravimetric Analysis (TGA)

Thermogravimetric analyses (TGA) were carried out using a TGA Discovery apparatus from TA Instruments^®^ (Guyancourt, France). The experiments were performed in platinum pans compatible with high temperatures, from 25 to 700 °C, under an oxygen dynamic gas flow of 25 mL min^−1^, with a balance purge of nitrogen at 25 mL min^−1^. Occasionally, the thermal decomposition was sought under nitrogen, at a similar flow, to highlight the influence of the atmosphere (inert vs. highly oxidative).

The calibration of mass was done by using mass standards and the calibration of the mass loss was performed with calcium oxalate. The calibration of temperature used the Curie point of nickel. The sample mass ranged between 1 and 10 mg. The experiments were performed in isothermal and anisothermal conditions.

Regarding anisothermal measurements, in addition to standard procedures using conventional heating rates, i.e., 5, 10, and 20 K min^−1^, specific protocols were designed for performing high-rate TGA, up to 500 K min^−1^, and TGA–FTIR (Guyancourt, France). These protocols are explained in the following sub-sections.

### 2.3. Preliminary Tests for the Measurements at High Rates

The impact of the heating rate on the calibration conditions, based on the determination of the Curie point of nickel, was previously investigated [35]. In a first step, the temperature correction was performed consecutively to the analysis of a nickel standard at the rate of 20 K min^−1^. Then, a subsequent run was performed at any rate ranging from 5 to 500 K min^−1^. For experiments performed at rates equal to or lower than 50 K min^−1^, the new experimental Curie point of nickel always coincided with the reference from the literature, showing the efficiency of the correction. However, for rates ranging from 100 to 500 K min^−1^, the experimental value differed from the reference. To take into account this divergence, the uncertainty on the characteristic temperatures was set to 10 °C, corresponding to the maximum difference that was reported [35].

The alignment of the sensor temperature with the theoretical time/temperature dependence was previously verified by plotting the temperature as a function of time for heating rates ranging from 5 to 500 K min^−1^ [35]. It was observed that, at standard rates, the sensor temperatures almost instantaneously aligned with the time/temperature dependence imposed by the experimental program, while a slight adjustment time was occasionally needed beyond 50 K min^−1^. This adjustment is set far below the temperature domain characteristic of the PPS and CF/PPS degradations, showing that the experimental conditions are acceptable to investigate the PPS pyrolysis.

### 2.4. Coupling of TGA Methods with Fourier-Transform Infrared Spectroscopy

The decomposition products were identified thanks to the TGA–FTIR coupling. A transfer line was connected between a TGA apparatus (Discovery model from Thermal Analysis^®^ Guyancourt, France) and a FT–IR spectrometer (Nicolet TM iSTM10 model from Thermoscientific^®^, Montigny-le-Bretonneux, France), equipped with a Helium–Neon laser source, a Ge/KBr beam splitter, and a deuterated triglycine sulphate (DTGS) pyroelectric detector. The database from the OMNIC^®^ 9.3. acquisition software allowed for the interpretation of the spectra.

## 3. Results and Discussion

### 3.1. General Features Regarding the Thermal Decomposition Under Oxygen

**Figure 1:**
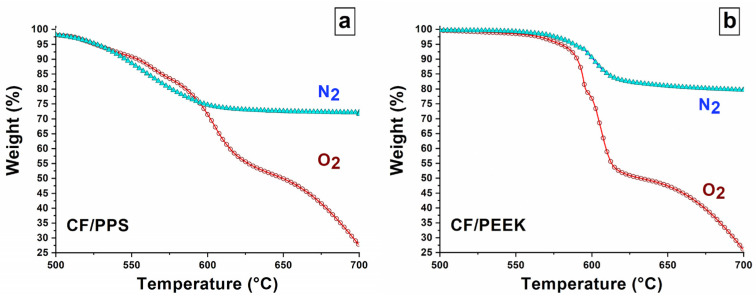
TGA experiments performed at 20 K min^−1^ under nitrogen and oxygen on (**a**) CF/PPS and (**b**) CF/PEEK.

Figure 1 presents the TGA responses recorded at 20 K min^−1^ for CF/PPS (Figure 1a) and CF/PEEK (Figure 1b) under nitrogen and oxygen. In both cases, the decomposition under nitrogen occurs by one single step, leading to a plateau that indicates the amount of mass residue, higher than 70% of the initial mass. As explained above, carbon fibers are stable under these thermal conditions. Thus, the TGA signature corresponds to the decomposition of the polymer matrix, from which the char is formed. In comparison, the analyses under oxygen reveal three steps (the dTG curve associated with the decomposition of CF/PPS is shown in the Supplementary Materials). The first causes a similar weight loss to the decomposition under nitrogen. Therefore, it is interpreted as the signature of char formation. The second step, situated immediately after, leads to a mass residue close to 50%, which approximately corresponds to the fiber content. Likely, this event marks the oxidative decomposition of the char. Eventually, the oxidative decomposition of the carbon fibers, evidenced during the third step, occurs at higher temperatures and is not terminated at the end of the experiment.

As expected, both composites show a greater sensitivity to oxidative conditions. Nevertheless, for CF/PPS, the char-formation signature is shifted toward higher temperatures in comparison with the TGA response obtained under nitrogen. This behavior is not observed for CF/PEEK. It may suggest that oxygen triggers chemical modifications within the PPS matrix that improve its thermal stability.

**Figure 2:**
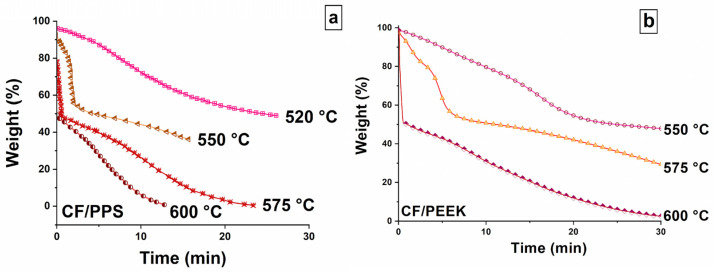
TGA experiments under oxygen performed in isothermal conditions on (**a**) CF/PPS and (**b**) CF/PEEK. The TGA responses are recorded at 520, 550, 575, and 600 °C for CF/PPS, and at 550, 575, and 600 °C for CF/PEEK.

Figure 2 shows the results of CF/PPS (Figure 2a) and CF/PEEK (Figure 2b) thermal decomposition under oxygen, in isothermal conditions, for various holding temperatures, covering the whole domain of the char formation. At several instances, a mass loss is recorded at the very beginning of the experiment. It is linked to the matrix, which decomposes when the composite is brought to the holding temperature. For example, when the decomposition is studied at 600 °C, the experiment starts with only 50% of the initial mass remaining, since the matrix has already volatilized. In these conditions, only the fiber-decomposition stage is followed. After a holding time of 30 min, the carbon fibers are fully volatilized.

By decreasing the holding temperature, it is possible to catch the three events identified during the anisothermal tests. When CF/PEEK is maintained at 575 °C, each step is clearly delimited, with the fiber decomposition corresponding to the broad signature recorded in the last twenty minutes of the experiment. In the case of CF/PPS, only the last stages of the char volatilization are caught at 575 °C. Thus, an additional experiment has been performed at 550 °C, making it possible to identify each step of the decomposition.

Eventually, the experiments performed in less aggressive conditions, i.e., 520 °C for CF/PPS and 550 °C for CF/PEEK, only reveal the matrix decomposition. For both materials, the char formation and its decomposition are not clearly separated. Thus, only one broad step is observed leading to a plateau that presumably precedes the fiber decomposition.

To summarize, oxygen can induce the full decomposition of the CF-reinforced composites independently of the polymer matrix selected. In addition to the char formation recorded under nitrogen, the TGA signatures also reveal two additional steps, supposedly indicative of the char oxidative decomposition and the fiber oxidative decomposition. On the other hand, for CF/PPS, oxygen may increase the intrinsic thermal stability of the matrix, as the char formation is shifted toward higher temperatures.

### 3.2. Decomposition Signatures for Fast Heating Rates

**Figure 3:**
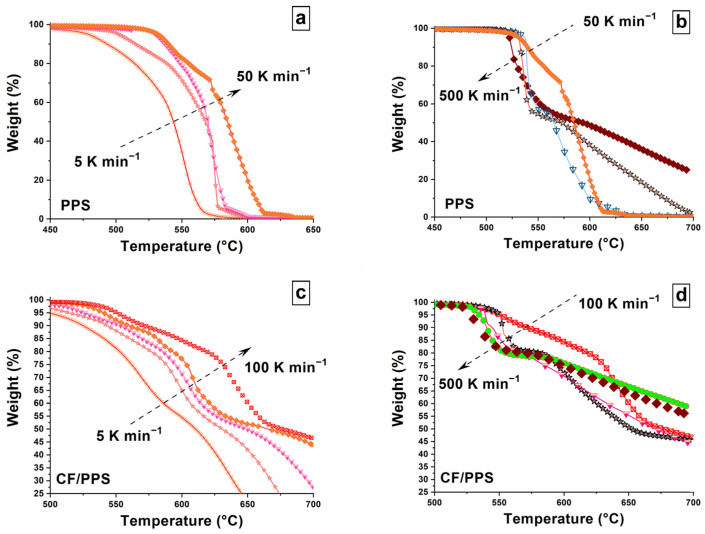
TGA experiments performed in an oxygen atmosphere on PPS and CF/PPS samples at various heating rates: (**a**) analyses of PPS at 5, 10, 20, and 50 K min^−1^; (**b**) analyses of PPS at 50, 100, 300, and 500 K min^−1^; (**c**) analyses of CF/PPS at 5, 10, 20, 50, and 100 K min^−1^; (**d**) analyses of CF/PPS at 100, 200, 300, 400, and 500 K min^−1^. For each material, the response at 500 K min^−1^ is highlighted with brown diamonds.

The impact of the applied rate on the TGA response of PPS and CF/PPS is shown in Figure 3. For clarity purposes, the series of curves is split into two categories, according to the heating rate. From 5 to 100 K min^−1^, the signature of PPS degradation shifts toward higher temperatures, with the heating rate increasing (Figure 3a); on the other hand it shifts toward lower temperatures when the heating rate increases from 100 to 500 K min^−1^ (Figure 3b). The same behavior is observed for CF/PPS, as presented in Figure 3c,d. These results are consistent with those previously obtained under nitrogen, from investigations led on several polymer systems [35], including PPS and CF/PPS. Thus, it is assumed that this reversal of the degradation shift systematically occurs, independently of the material and the atmosphere.

Interestingly, several pieces of information can also be extracted from the residual mass. As shown in Figure 3a, PPS matrix decomposition is total when the ramp is executed at rates ranging from 5 to 50 K min^−1^. However, by increasing the heating rate, up to 500 K min^−1^ (Figure 3b), the time allowed for the char decomposition is shortened. Thus, at 300 K min^−1^, this event is delayed, starting at 600 °C, and it is even incomplete at 500 K min^−1^ (the residue weight is 30% of the initial weight). Similar observations can be made for CF/PPS. From heating rates ranging from 5 to 20 K min^−1^, the three decomposition steps are recorded, whereas the carbon fiber does not decompose at rates equal to 50 and 100 K min^−1^ (Figure 3c). The char decomposition itself is even hindered at 400 and 500 K min^−1^ (Figure 3d). The decomposition under oxygen, although possible, is obviously a time-consuming process. This aspect must be considered when investigating the impact of the rate on the decomposition mechanisms, as the char formation is the only event that fully proceeds independently of the rate.

**Figure 4:**
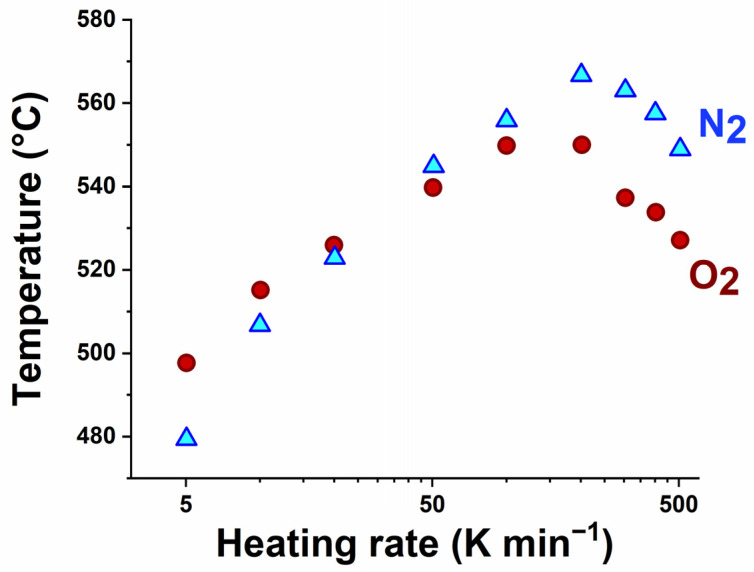
Impact of the heating rate on the onset of char formation for experiments performed under oxygen and nitrogen.

The resulting profiles of the onset decomposition temperature vs. the heating rate are presented in Figure 4. Only the onset for the char formation is considered there, since the occurrence of the three steps is conditioned by the rate of analysis. For comparison purposes, the results obtained under nitrogen are added. Similar bell-shape trends are observed independently of the atmosphere. This behavior was tentatively interpreted as the combination of two effects [35,37]. The mass effect, as reported by Vanden Poel et al. [37], is related to the thermal conductivity. By increasing the heating rate, the thermal lag between the sample temperature and the sensor temperature increases, which continuously shifts the decomposition signature toward higher temperatures. Owing to this effect, it was previously observed that the experimental data gathered from investigations on several polymers (HDPE, PET, EcoPaXX, PLA, PA6, and PPS) are aligned from 5 to 100 K min^−1^ [35], when presented in a normalized scale (T_d max_–T_d 5K_, with T_d 5K_ being the reference temperature at which the decomposition rate is the fastest when the heating rate is equal to 5 K min^−1^). The second effect, linked to the thermal lag between both the furnace and sensor temperatures, causes the decomposition signature to shift toward lower temperatures, for heating rates ranging from 100 to 500 K min^−1^. This rate effect is systematically observed, also with nickel, but its magnitude seems to be sample dependent [35].

It is worth mentioning that, at the lowest rate, i.e., 5 K min^−1^, the decomposition under oxygen is delayed for both PPS and CF/PPS, in comparison with the decomposition under nitrogen, as previously observed in Figure 1a. While the uncertainty regarding the decomposition temperature is expected to be close to 10 °C at high heating rates, the measurement should be more accurate at 5 K min^−1^. Thus, this result could be the signature of a chemical process, contributing to increases in the thermal stability of the PPS matrix under oxygen. Thermal degradation is often presented as a competition between chain scission and crosslinking mechanisms. While chain scissions fragilize the polymer, crosslinking reactions can improve its thermal stability. Therefore, the oxidative degradation of PPS should induce crosslinking reactions that are absent, or less favored, in a nitrogen atmosphere.

This assumption is supported by several studies. Gros et al. [38] identified intermolecular branching within PPS consecutive to an annealing at 250 °C under air. Yan et al. [39] have investigated the structural evolution of PPS during thermal degradation in nitrogen and oxygen atmospheres by rheological measurements. They showed that the gel content increases substantially with temperatures elevating, in the case of a thermal treatment under oxygen, while the apparition of insoluble components is a marginal event in the case of a thermal treatment under nitrogen. Furthermore, Vieille et al. [7] evidenced from dynamic mechanical analysis that an annealing under air for 1271 h at 230 °C induces a shift of the glass transition signature by more than 50 °C, and a brutal increase of the elastic modulus in the rubbery state. Eventually, Martin et al. [40] reported a significant increase in cross-linkage, observed after a long induction period, for aging performed under air at 753 K. This set of findings suggests that the chain scission is the predominant mechanism of thermal degradation under nitrogen, whereas the temperature elevation also triggers crosslinking when it occurs in an oxidative atmosphere.

**Figure 5:**
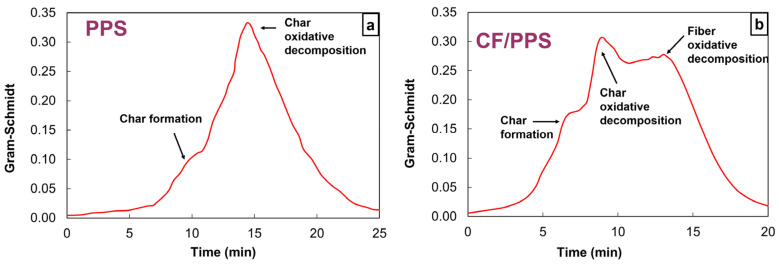
Gram–Schmidt signal obtained from TGA–FTIR experiments performed under oxygen at 10 K min^−1^ on (**a**) PPS and at 20 K min^−1^ on (**b**) CF/PPS. The contributions characteristics of the decomposition steps are highlighted by arrows. The data are presented in a normalized timescale.

To investigate further the decomposition mechanisms, TGA–FTIR experiments have been performed at 10 K min^−1^ on PPS and at 20 K min^−1^ on CF/PPS (Figure 5). The main advantage of TGA–FTIR relies on its specificity. While MS detectors are useful to identify a whole bunch of decomposition products, FTIR makes it possible to accurately track the degassing of one product showing a strong infrared signature, thanks to a direct comparison with theoretical spectra. Besides, not only the emission of the product is recorded, but also its relative concentration can be estimated during the whole decomposition, which makes it possible to highlight successive mechanisms when relevant. The raw TGA–FTIR signature is the Gram–Schmidt signal, which provides, for a given time, the sum of the entire IR absorbance for all wavenumbers. The Gram–Schmidt signal helps to set the interval within which the analysis of the detected decomposition gases should be preferentially performed. Its maximum is related to the maximum rate of the degradation. As several contributions appear in the Gram–Schmidt response, it is suspected that the decomposition occurs through multiple stages. For PPS, two distinct contributions are identified (Figure 5a), while the decomposition of CF/PPS leads to three events (Figure 5b). Considering that the Gram–Schmidt signals of both PPS and CF/PPS exhibit only one contribution when their decomposition occurs in a nitrogen atmosphere [35], it is assumed that the additional contributions should be attributed to oxidative mechanisms. Thus, the first peak would relate to the char formation, while the second one would characterize the oxidative decomposition of the char. Carbon fibers being more prone to resist thermal oxidative decomposition than the matrix, the third peak, only observed for CF/PPS, would indicate the oxidative decomposition of the fibers.

**Figure 6:**
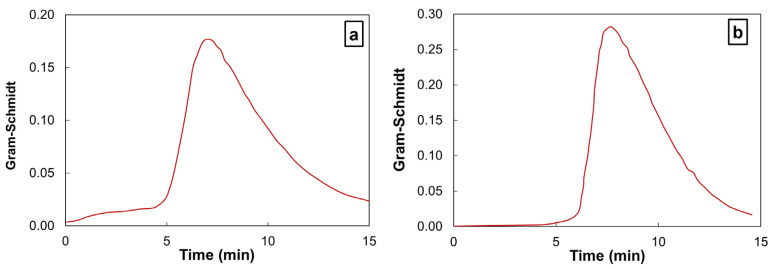
Gram–Schmidt signal obtained from TGA–FTIR experiments performed under oxygen at 100 K min^−1^ on (**a**) PPS and (**b**) CF/PPS. The data are presented in a normalized timescale.

To investigate the impact of the heating rate on the resulting decomposition mechanisms, it is required to select rates at which each event of interest proceeds in its entirety. The rates of 400 and 500 K min^−1^ are seemingly too high because they do not allow for the char oxidative decomposition. Therefore, the highest rate for TGA–FTIR investigations has been set to 100 K min^−1^. As shown in Figure 6, the increase of the heating rate up to 100 K min^−1^ induces a modification of the Gram–Schmidt profile. The previously recorded events merge into one broad signature. It is worth noting that the Gram–Schmidt profile is identical for PPS and CF/PPS. This result was expected because the carbon-fiber decomposition does not occur at 100 K min^−1^.

Considering the merger of the contributions in the Gram–Schmidt signal, it is not ascertained that the stages of decomposition are clearly spaced out in time for quick elevations of temperature. To identify the decomposition products, an infrared spectrum has been recorded at regular intervals of the Gram–Schmidt signature. Thus, it is possible to analyze the decomposition products characteristic of each stage of the decomposition, which can be clearly identified at standard rates, and then compare the results with those obtained at 100 K min^−1^. For each probed area of the Gram–Schmidt signal, the nature of the decomposition products has been sought, by verifying the level of agreement between the infrared spectrum from the experiment and the theoretical signature expected for given molecules according to databases.

**Figure 7:**
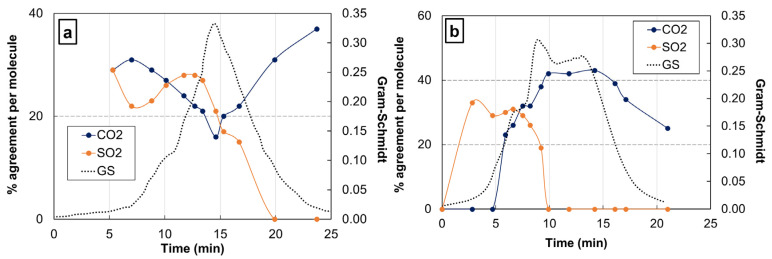
TGA–FTIR agreement percentage between the experimental spectra of (**a**) PPS and (**b**) CF/PPS thermal decomposition gases obtained at 10 K min^−1^ and 20 K min^−1^ respectively, under oxygen, and the reference spectra of carbon dioxide and sulfur dioxide. The time boundaries of the degradation process are deduced from the Gram–Schmidt signal. The data are presented in a normalized timescale.

The thermal oxidative decomposition of PPS has been studied by Kumaga et al. [41]. The main decomposition products that have been identified are water, carbon dioxide, sulfur dioxide, and small aromatic molecules containing sulfur atoms. We focus on sulfur dioxide (SO_2_) and carbon dioxide (CO_2_) there, since it is assumed that the amount of emitted SO_2_ should be high during the char formation, but almost insignificant during the oxidative decomposition of both the char, which is a carbon residue, and the carbon fibers. Thus, in Figure 7, the agreement level between experimental spectra and spectra from the databases is presented, for TGA–FTIR experiments performed at 10 K min^−1^ on PPS (Figure 7a) and at 20 K min^−1^ on CF/PPS (Figure 7b). Consistenct with our expectations, SO_2_ is a major component among the identified products for the early stages of decomposition. Then, it vanishes, while the content of emitted CO_2_ remains stable during the whole decomposition.

**Figure 8:**
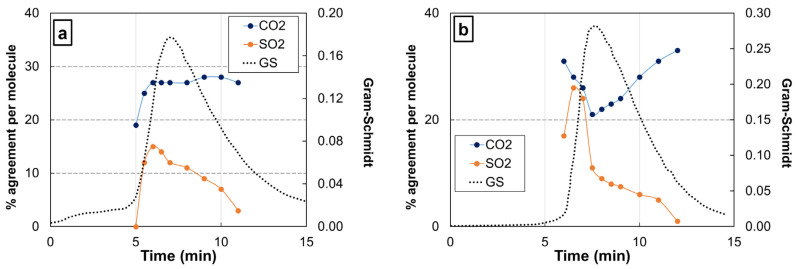
TGA–FTIR agreement percentage between the experimental spectra of (**a**) PPS and (**b**) CF/PPS thermal decomposition gases at 100 K min^−1^ under oxygen, and the reference spectra of carbon dioxide and sulfur dioxide. The time boundaries of the degradation process are deduced from the Gram–Schmidt signal. The data are presented in a normalized timescale.

The agreements resulting from high-rate TGA–FTIR analyses are presented in Figure 8. Since the carbon fibers do not decompose at 100 K min^−1^, according to Figure 3d, the results obtained for PPS (Figure 8a) and CF/PPS (Figure 8b) should share similarities. Indeed, it is observed in both cases that the selectivity regarding the quantity of emitted products is less apparent. Carbon dioxide is the main component among the identified products, independently of the Gram–Schmidt domain, whereas sulfur dioxide is still detected but in lesser concentrations.

Previous investigations have highlighted the impact of the heating rate on the decomposition mechanism. Li et al. [42] reported that the fast pyrolysis of algal waste produces 50% more carbon dioxide than slow pyrolysis. Efika et al. [43], investigating waste wood pellets, reported changes of the solid/liquid/gas product ratio with the increase in heating rate. Regarding PPS and CF/PPS, the study of the thermal decomposition under nitrogen has revealed that the random chain–scission mechanism progressively replaces the depolymerization when the heating rate increases [35]. In the present study, there is no such mechanism reversal suspected. On the other hand, it is assumed that the temperature window, within which oxygen preferentially triggers the emission of SO_2_, is thin. Thus, it is quickly bypassed when the heating rate increases.

## 4. Conclusions

Thermo-oxidative decomposition is a critical risk for the composites used in the aeronautic field, which is faced when some components catch fire in the aircraft. To mimic the sudden elevation of temperature, the decomposition of carbon fiber–reinforced poly(phenylene sulfide) has been studied by performing thermogravimetric analyses at high rates, up to 500 K min^−1^. Working under oxygen allowed us to seek the material response in a highly reactive environment.

Depending on the applied rate, the thermogravimetric signature changes. In standard conditions, three stages of decomposition are observed, respectively corresponding to the char formation, the char oxidative decomposition, and the fiber decomposition. This result is specific to oxidative environments, as the thermogravimetric signature under nitrogen only shows the char formation. By increasing the heating rate, the time allowed for the decomposition is not sufficient. Therefore, it is observed that the fibers do not decompose for rates equal to or higher than 50 K min^−1^, while the char decomposition is hindered at 400 and 500 K min^−1^. For isothermal experiments, the thermogravimetric signature depends on the holding temperature. It can either highlight the fiber decomposition, the char formation, or even the three stages.

It is worth mentioning that the high–rate investigations are designed to catch the mechanisms in the early stages of decomposition. Indeed, the procedure is restricted to a ramp that stops at 700 °C, while the thermal solicitation is continuous during a fire. This means that the fiber decomposition, which is not recorded at 500 K min^−1^, will certainly occur by maintaining the material under the same thermal oxidative conditions.

By analyzing the decomposition products for various experimental conditions, it is shown that the emission of sulfur dioxide is predominant during the early stages of decomposition and stops during the char oxidative decomposition in favor of the emission of carbon dioxide. By increasing the heating rate, the stages merge into one global process and the ratio between the decomposition products changes accordingly.

## Data Availability

The original contributions presented in the study are included in the article/Supplementary Materials, further inquiries can be directed to the corresponding author.

## Supplementary Materials

The following supporting information can be downloaded at: https://www.mdpi.com/article/10.3390/polym17111560/s1, Figure S1: Time/temperature profile related to the decomposition of PPS under oxygen at 500 K min−1; Figure S2: The main steps of decomposition highlighted by the DTG curve for CF/PPS analyzed at 20 K min^−1^.
