# Supplementary material for: Oxidative Decomposition of Poly(phenylene sulfide) Composites Under Fast Elevation of Temperature
Source: Polymers (Basel). 2025 Jun 3;17(11):1560. doi: 10.3390/polym17111560 (PMC12157743; doi:10.3390/polym17111560)
Supplement: Supplementary file 1 [file polymers-17-01560-s001.zip › polymers-3647685-supplementary.pdf]

polymers

Supporting Information

Oxidative Decomposition of Poly(phenylene sulfide) Composites Under Fast Elevation of Temperature

Aurélie Bourdet, Yann Carpier, Eric Dargent, Benoit Vieille and Nicolas Delpouve *

INSA Rouen, UNIROUEN, Normandie Univ, CNRS, GPM UMR6634, 76000 Rouen, France; aurelie.bourdet@ensam.eu (A.B.); yann.carpier@latecoere.aero (Y.C.); eric.dargent@univ-rouen.fr (E.D.); benoit.vieille@insa-rouen.fr (B.V.)

* Correspondence: nicolas.delpouve1@univ-rouen.fr; Tel.: +33-(0)232955165

Figure S1. Time/temperature profile related to the decomposition of PPS under oxygen at 500 K min-1.
Figure S2. The main steps of decomposition highlighted by the DTG curve for CF/PPS analyzed at 20 K min-1.

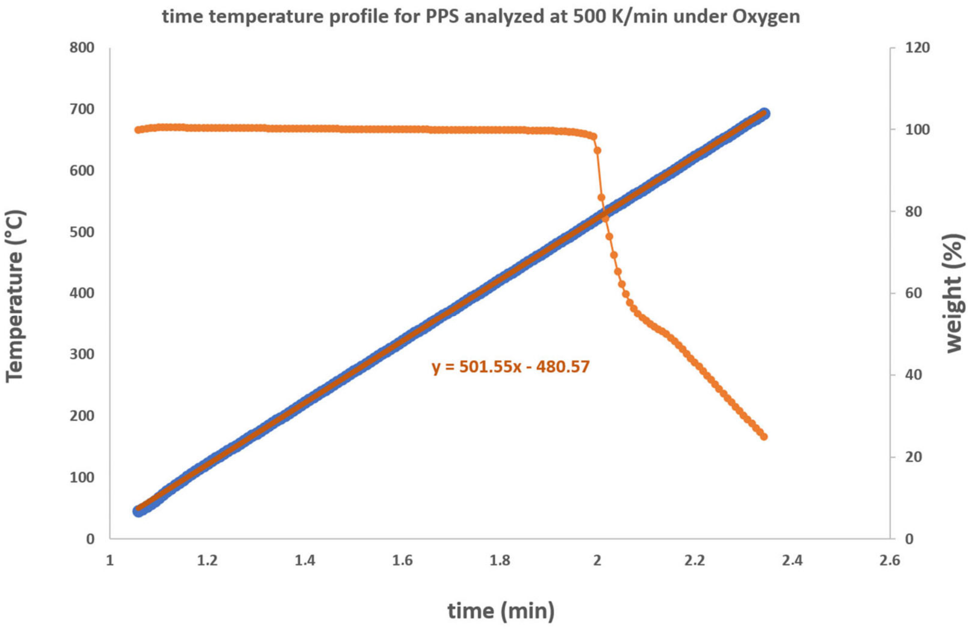

Figure S1. Time/temperature profile related to the decomposition of PPS under oxygen at 500 K min^{-1}. The experimental data properly follow the temperature program in the decomposition range.

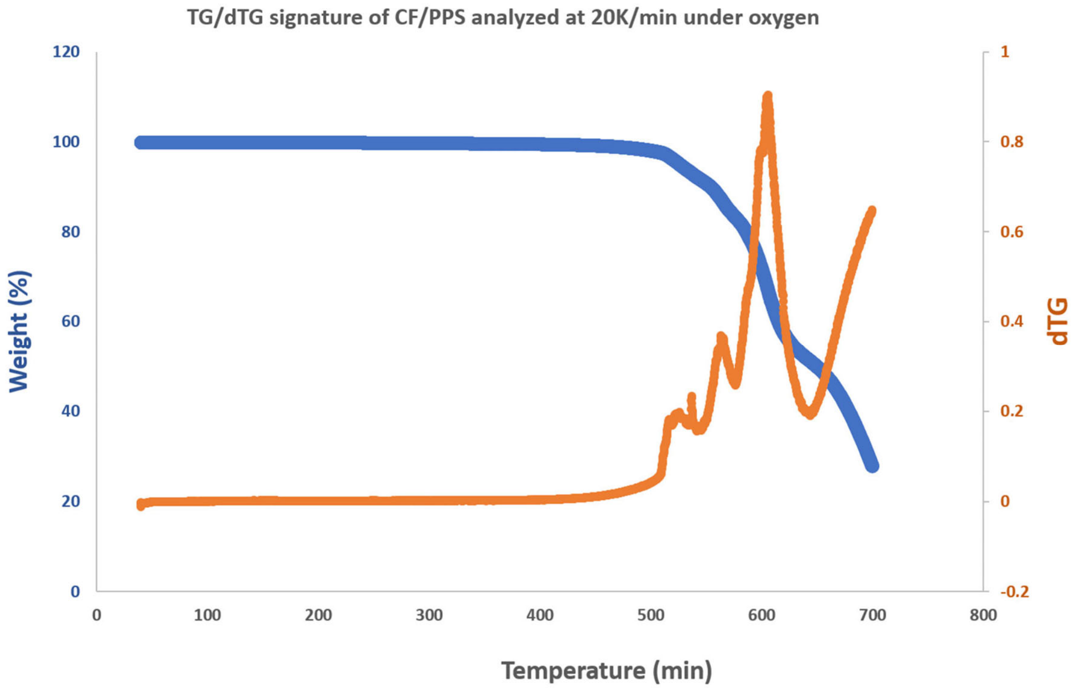

Figure S2. The main steps of decomposition highlighted by the DTG curve for CF/PPS analyzed at 20 K min^{-1}. The last and incomplete step corresponds to the fiber oxidative decomposition. It is preceded by the char formation then the char oxidative decomposition.
